# Supplementary material for: Upregulation of Antioxidative Gene Expression by Lasia spinosa Organic Extract Improves the Predisposing Biomarkers and Tissue Architectures in Streptozotocin-Induced Diabetic Models of Long Evans Rats
Source: Antioxidants (Basel). 2022 Dec 2;11(12):2398. doi: 10.3390/antiox11122398 (PMC9774390; doi:10.3390/antiox11122398)
Supplement: Supplementary file 1 [file antioxidants-11-02398-s001.zip › antioxidants-2045034-supplementary.pdf]

Supplementary files

Table S1: Preliminary phytochemical status of different extracts of *Lasia spinosa*

Compound	Name of test	Observation	Result LSML
**Alkaloids**	Mayer's test	White or creamy white precipitate	+
	Wagner's test	Brown or deep brown precipitate	+
	Dragendorff's test	Orange or orange-red color	+
**Flavonoids**	NaOH test	Canary yellow color	+
	Lead (II) acetate test	Bulky white precipitate	+
**Steroids**	Liberman test	Blue to blood red color	+
	Salkowski's test	Reddish brown color	+
**Tannins**	General color test	Green-black to blue color	+
**Saponins**	Frothing test	Formation of stable foam.	-
**Phlobatannins**	General color test	Red precipitate	-
**Glycosides**	Keller-killiani test	Bluish -green ring or violet ring	-
**Carbohydrates**	Molish test	A deep violate color formed at the junction of the two layers	+
	Benedict test	green color	+
**Proteins**	Biruet's test	violet or pink color	+
	Ninhydrin test	Purple or dark Purple color	+

Here, (+) sign indicates presence and (–) sign indicates absence. LSML- Methanolic leaf extract of *Lasia spinosa*
